# The expression of BRCA1, P53, KAI1, and Nm23 in ovaries of BRCA1 mutation carriers after prophylactic adnexectomy

**DOI:** 10.1007/s00404-013-2825-9

**Published:** 2013-04-04

**Authors:** J. Markowska, J. Bar, R. Mądry, I. Słomska, M. Mardas, J. P. Grabowski

**Affiliations:** 1 Division of Gynecology, Department of Oncology, Poznan University of Medical Sciences, Poznan, Poland; 2 Department of Clinical Immunology, Wrocław Medical University, Wrocław, Poland; 3 Department of Gynecology and Gynecological Oncology, Kliniken-Essen-Mitte, Henricistr. 92, 45136 Essen, Germany

**Keywords:** BRCA1, Prophylactic adnexectomy

## Abstract

**Introduction:**

High mortality rate, absence of reliable methods for early diagnosis and poor prognosis of advanced ovarian cancer prompted to investigate the role of prophylactic oophorectomy in BRCA1 mutation carriers as well as evaluate the expression of BRCA1, p53, Nm23, and KAI1 proteins in ovarian tissue from these patients.

**Materials and methods:**

Ovaries from BRCA1 mutation carriers underwent prophylactic adnexectomy and control group of patients were operated from other than cancer reasons. The expression of selected proteins was studied using immunohistochemical staining. The intensity of immunostaining and the number of tumor cells showing the reaction for selected proteins were analyzed.

**Results:**

We have analyzed ovarian tissues from 18 BRCA1 mutation carriers and 11 women included in control group. Positive expression of BRCA1 protein was presented in 83.3 % cases in BRCA1 mutation carriers and in 72.7 % in the control group (*p* > 0.05). Positive expression of p53 protein was observed, respectively, in 27.8 vs. 36.4 % (*p* > 0.05), Nm23 protein 77.7 vs. 90.9 % (*p* > 0.05), and KAI1 in 72.2 vs. 72.7 % (*p* > 0.05). Mean percent of tumor cells that showed the reaction for selected proteins as well as the intensity of immunostaining for all analyzed proteins seems to be lower in BRCA1 mutation carriers.

**Conclusions:**

However, any significant differences between study group and control group have not been found; there were similar trends showing reduced expression of studied proteins in BRCA1 mutation carriers.

## Introduction

Epithelial ovarian cancer is often referred as the “silent killer” according to its asymptomatic clinical pattern, mostly diagnosed in its advanced stages [1]. Approximately 10 % of ovarian cancer cases are attributed to the inheritance of a BRCA1 or 2 mutation [2]. The lifetime risk of ovarian cancer in patients with a BRCA1 mutation is between 40 and 60 % [1, 3] compared with the general population risk of 1.8 % [4]. Due to high mortality rate, absence of reliable methods for early diagnosis and bad prognosis of advanced stages of ovarian cancer the role of prophylactic adnexectomy remain the therapy of choice [2, 4]. Especially, assuming significant lifetime risk reduction for developing ovarian cancer and a low probability of peritoneal dissemination according to three cohort studies in patients undergoing bilateral adnexectomy [2].

BRCA1 was identified and cloned in 1994 [1]. This gene is localized on chromosome 17 (17q21) and codes large proteins that consist of 1,863 amino acids. BRCA1 protein has a large number of functions, including regulation of transcription, embryological development, immune modulation, cell cycle control, and DNA repair [5, 6]. The loss of the last function leads to accumulation of unrepaired somatic mutations what may result in development of ovarian cancer [5].

The p53 protein has an important biologic function because it interferes in the response to DNA damage. The protein accumulates in the cell nucleus and is activated as a transcription factor in response to DNA damage, hypoxia, and other genotoxic stresses. It can block the cell cycle in G1, allowing the cell to repair genomic damage, or it can induce apoptosis. Loss of p53 function plays a central role in the development of cancer. In fact, p53 is mutated in 40–80 % of epithelial ovarian cancers [6, 7]. It is known that BRCA1 interacts with p53 in the normal tumor suppressor pathway. A higher prevalence of p53 mutations has been identified in the BRCA related tumors than in sporadic ovarian cancer [5].

A metastasis suppressor gene on human chromosome 11p11.2 that encodes a glycoprotein of the transmembrane four superfamily (TM4SF) is the highly glycosylated protein KAI1 (CD 82). Although the precise biological functions of TM4SF molecules are not fully elucidated, several studies have shown involvement in cell growth, adhesion, and motility. This protein is physiologically expressed in a variety of tissues and was reported to be downregulated in several types of human cancer, including cancer of the ovary, cervix, prostate, lung, breast, bladder, colon, and pancreas [8–10].

Nm23 gene product has activity of nucleoside diphosphate kinase (NDPK). Gene has been reported to be involved in cell proliferation and differentiation. Reduced expression of the Nm23 gene is implicated in the metastatic progression of various cancers: breast, ovary, cervix, liver, colon, gastric, and melanoma. Although expression of Nm23 is divergent in various malignant tumors, its downregulated expression seems to be related with increased metastatic potential in most carcinoma types [11–13].

The aim of this study was to investigate the expression of BRCA1 protein as well as p53, Nm23, and KAI1 proteins associated with tumor metastatic progression in ovarian tissue removed during prophylactic adnexectomy in BRCA1 mutation carriers.

## Materials and methods

Ovarian tissue was collected from BRCA1 mutation positive patients during prophylactic bilateral adnexectomy. As a control group, samples from patients operated in our Department from other than cancer reason were evaluated. All patients in control group were not BRCA mutation carriers.

Immunohistochemical staining for analyzed proteins was performed on frozen 5-μm tissue sections using the Universal DakoCytomation LSAB + Kit, Peroxidase procedure (LSAB + Kit: HRP, Dako, Copenhagen, Denmark) and following primary antibodies: monoclonal antibody anti-p53 protein (clone DO-7) (Novocastra, Newcastle, UK), monoclonal antibody anti-nm23 protein (Novocastra), monoclonal antibody anti-KAI 1 (G-2) (Santa Cruz, USA), and polyclonal antibody anti-BRCA1 (C-20) protein (Santa Cruz). Nonspecific tissue and endogenous peroxidase reactivity were blocked with 10 % BSA (Bovine serum albumin) and 3 % H_2_O_2_, respectively. Tissue specimens were incubated with primary antibodies 60 min at room temperature. Following washing with 0.1 M Tris-buffer, pH 7.4 (TBS), the tissue specimens were incubated with secondary biotinylated rabbit antibody, anti-mouse/rabbit IgG (Dako, Denmark) and with streptavidin horseradish peroxidase conjugated (Dako) both for 15 min at room temperature. After washing with TBS, the antigen–antibody reaction was visualized by DAB (3,3 diaminobenzidine) (Dako, Denmark) as a chromogen (8 min, room temperature). Sections were counterstained with hematoxylin and mounted. The incubation buffer (TBS) without primary antibodies was used as negative control. The internal positive controls were used.

The evaluation of immunohistochemical data was performed as following. Sections were scored semiquantitatively, taking into account the intensity of immunostaining and the number of tumor cells showing the reaction for p53, Nm23, KAI1, and BRCA1 protein. The cases were score as positive for p53 protein expression when, more than 15 % of the tumor cells revealed nuclear immunopositivity. KAI1, Nm23, and BRCA1 expression was assessed positive if more than 10 % of tissue section showed immunostaining.

Statistical analysis has been performed using STATISTICA 6.0.

## Results

Overall, ovarian tissues from 29 patients were examined. Studied group included 18 (62 %) patients with BRCA1 mutation (mean age 50.1 ± 8.2). Among those, 10 women had previous history of breast cancer. Control group included 11 (38 %) patients operated from other than cancer reasons (mean age 51.6 ± 10.2). We did not observe any postoperative complications.

In one woman from a study group, high-grade serous ovarian cancer was diagnosed. This patient has not suffered from a breast cancer in the past. After completing staging surgery, tumor was classified as FIGO IC.

**Fig. 1:**
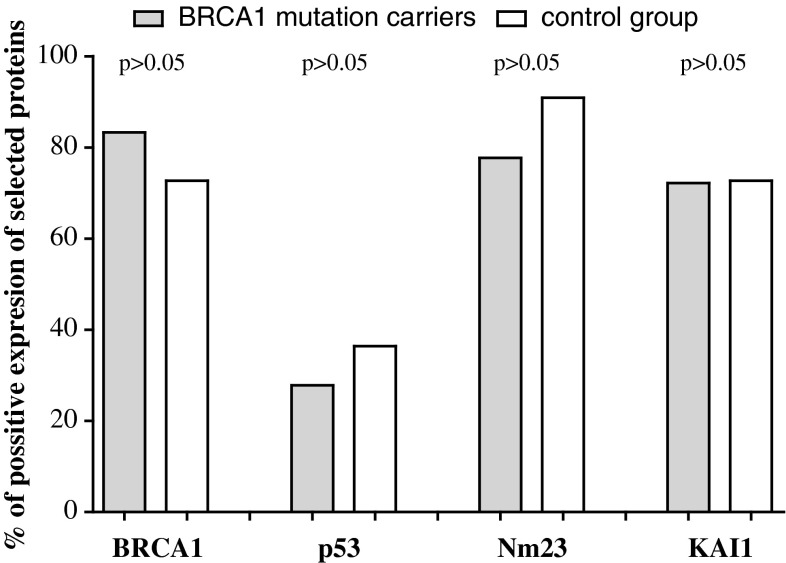
The percent of positive expression of BRCA1, p53, Nm23, and KAI1 proteins in BRCA1 mutation carriers and control group

**Fig. 2:**
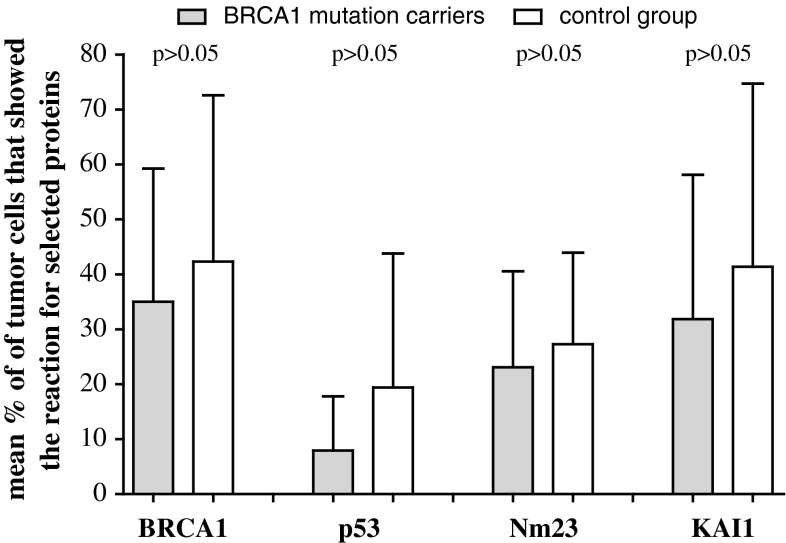
Mean percentage value of the tumor cells showing the reaction for BRCA1, p53, Nm23, and KAI1 proteins

**Fig. 3:**
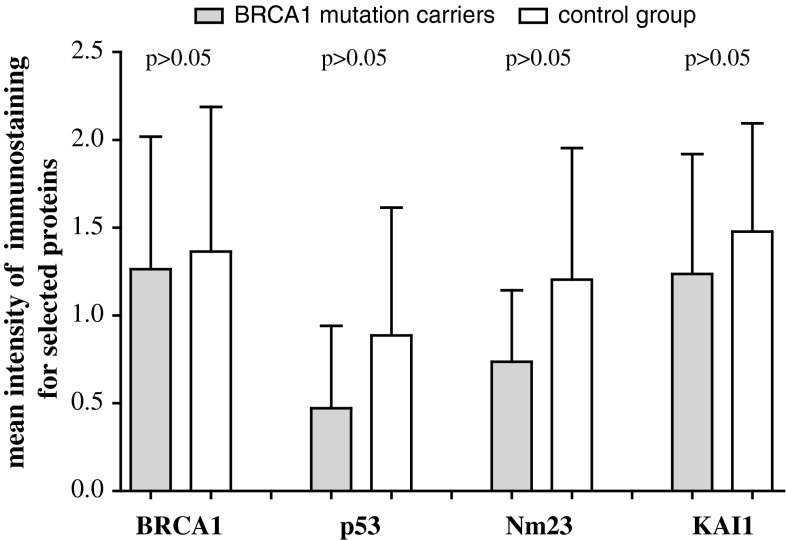
Mean values of the intensity of immunostaining (ou/μm^2^) for BRCA 1, p53, Nm23, and KAI1 proteins

As shown in Fig. 1 expression of BRCA1 protein in ovarian tissue was presented in 83.3 % (15 pat.) BRCA1 mutation carriers and in 72.7 % (8 pat.) patients in control group. Positive expression of p53 protein was observed, respectively, in 27.8 % (5 pat.) vs. 36.4 % (4 pat.), of Nm23 protein 77.7 % (14 pat.) vs. 90.9 % (10 pat.), and of KAI1 in 72.2 % (13 pat.) vs. 72.7 % (8 pat.) cases. Mean percentage values of tumor cells showing the reaction for BRCA1, p53, Nm23, and KAI1 proteins are presented in Fig. 2. It has been identified that mean cell percentage in which expression of BRCA1 protein was identified in BRCA1 mutation carriers which reached 33.3 % (6 pat.) and in the control group 45.4 % (5 pat.), respectively. p53 protein was detected in 5.6 % (1 pat.) vs. 18.1 % (2 pat.), Nm23 protein 22.2 % (4 pat.) vs. 27.3 % (3 pat.), and KAI1 33.3 % (6 pat.) vs. 45.4 % (5 pat.) of cells, respectively. Mean values of the intensity of immunostaining for all analyzed proteins are presented in Fig. 3. For BRCA1 protein, mean value of intensity of immunostaining in BRCA1 mutation positive group was 1.26 ou/μm^2^ and in the control group 1.36 ou/μm^2^. Accordingly, for p53 protein 0.47 vs. 0.88 ou/μm^2^, for Nm23 protein 0.73 vs. 1.20 ou/μm^2^, and for KAI1 1.24 vs. 1.47 ou/μm^2^.

The group of BRCA1 mutation carriers has been additionally evaluated according to medical history of breast cancer. Positive expression of BRCA1 protein was present in nine patients (90.0 %) in the group with breast cancer and in six patients (75.0 %) in BRCA1 mutation carriers without breast cancer. Positive expression of p53 protein was observed, respectively, in 4 cases (40.0 %) vs. 1 case (12.5 %), of Nm23 protein 8 (80. %) vs. 6 (75.0 %), and of KAI1 in 7 (70. %) vs. 6 (75.0 %). Mean percentage values of cells showing the reaction for BRCA1 were 30.0 % in breast cancer group vs. 37.5 % in BRCA1 mutation carriers without breast cancer. Mean percentage values of the tumor cells showing the reaction for p53 protein were, respectively, 0 vs. 12,5 %, for Nm23 20.0 vs. 25.0 %, and for KAI1 30.0 vs. 37.5 %. Mean values of the intensity of immunostaining for BRCA1 protein were 1.0 ou/μm^2^ in the group with breast cancer versus 1.3 ou/μm^2^ in BRCA1 mutation carriers only without breast cancer. Mean values of the intensity of immunostaining for p53 protein were, respectively, 0.41 vs. 0.53 ou/μm^2^, for Nm23 0.72 vs. 0.75 ou/μm^2^, and for KAI1 1.1 vs. 1.4 ou/μm^2^.

Three types of BRCA1 mutations were identified within the patients in our study. We have not found differences in patients showing expression of BRCA1, p53, KAI1, and Nm23 proteins regarding the type of mutation and breast cancer history (Table 1). According to percent of cells showing expression of studied genes, we have observed lower values in patients with breast cancer history in all types of BRCA1 mutations (Table 2). In Table 3 are presented mean values of the intensity of immunostaining that did not present significant differences.

**Table 1:** BRCA1 mutations carriers showing expression of BRCA1, p53, KAI1, and Nm23 proteins

BRCA1 mutation type	BRCA1	p53	KAI1	Nm23
ex 20 (5382 ins C)				
All (*n* = 9)	8 (88.9 %)	3 (33.3 %)	6 (66.7 %)	7 (77.8 %)
BC (*n* = 6)	6 (100 %)	2 (33.3 %)	4 (66.7 %)	4 (66.7 %)
ex 5 (300 T/G)				
All (*n* = 7)	5 (71.4 %)	1 (14.3 %)	5 (71.4 %)	5 (71.4 %)
BC (*n* = 3)	2 (66.7 %)	0 (0 %)	3 (100 %)	3 (100 %)
ex 11.17 (4153 del A)				
All (*n* = 2)	2 (100 %)	1 (50 %)	2 (100 %)	2 (100 %)
BC (*n* = 1)	1 (100 %)	1 (100 %)	1 (100 %)	1 (100 %)

*BC* breast cancer history, *n* number of patients

**Table 2:** Mean percentage value of cells showing the reaction for BRCA1, p53, KAI1, and Nm23 in BRCA1 mutation carriers indicating the type of BRCA1 mutation and the breast cancer history

BRCA1 mutation type	BRCA1 (%)	p53 (%)	KAI1 (%)	Nm23 (%)
ex 20 (5382 ins C)				
No BC (*n* = 3)	38.3	8.9	40.8	25.1
BC (*n* = 6)	47	6.7	38.3	21.8
ex 5 (300 T/G)				
No BC (*n* = 4)	36.3	7.5	46.7	7.5
BC (*n* = 3)	33.3	3.3	18.8	31.7
ex 11.17 (4153 del A)				
No BC (*n* = 1)	30	11.4	47.5	32.5
BC (*n* = 1)	20	15	15	25

*BC* breast cancer history, *n* number of patients

**Table 3:** Mean values of the intensity of immunostaining (ou/μm^2^) for BRCA1, p53, KAI1, and Nm23 in BRCA1 mutation carriers indicating the type of BRCA1 mutation and the breast cancer history

BRCA1 mutation type	BRCA1	p53	KAI1	Nm23
ex 20 (5382 ins C)				
No BC (*n* = 3)	0.67	0.58	1.5	1
BC (*n* = 6)	1.38	0.42	1.21	0.63
ex 5 (300 T/G)				
No BC (*n* = 4)	1.31	0.31	0.75	0.56
BC (*n* = 3)	1	0.33	1.58	0.75
ex 11.17 (4153 del A)				
No BC (*n* = 1)	1.75	1	1.75	0.75
BC (*n* = 1)	2.5	1	1.75	1.25

*BC* breast cancer history, *n* number of patients

## Discussion

Recent progress in our understanding of familiar ovarian cancer has led to significant changes in the day-to-day practice of surgical pathology [5]. Three cohort studies have identified a risk reduction with prophylactic bilateral adnexectomy in women with germ line BRCA1 or 2 mutations by comparing the incidence of ovarian cancer in the control group to the incidence of primary peritoneal carcinoma in the prophylactic adnexectomy group. Assumed analysis showed significant lifetime risk reduction for developing ovarian cancer and a low probability of peritoneal cancer in those undergoing surgery [2]. The development of cancer in BRCA germline mutation carriers occurs only if there is subsequent inactivation of the remaining wild-type BRCA allele on the opposite chromosome in addition to the pre-existing BRCA germline mutation [5].

In the study of Wang et al. [14], decreased expression of BRCA1 was found in 16 % of benign tumors, 38 % of borderline tumors, and 72 % of carcinomas. These results suggest that downregulation of BRCA1 protein play an important role in the development ovarian cancers [14]. In our study, positive expression of BRCA1 protein was observed 83.3 % of BRCA1 mutation carriers in comparison to 72.7 % in control group; however, the mean percentage value of the tumor cells showing the reaction for BRCA1 protein in BRCA1 mutation carriers was reduced in comparison to control group. The same results were observed in the intensity of immunostaining. Patients with history of breast cancer have higher expression rate of BRCA1; however, regarding to mutation of this gene function its product is impaired.

Loss of p53 function plays a central role in the development of cancer. The biological consequence of a missense mutation is enhancement of p53 stability and accumulation in the tumor cell nucleus [7]. The p53 alterations indisputably occur more often in BRCA1-associated tumors than in sporadic breast or ovarian tumors. This implies that loss of p53 function is a critical event in the molecular pathogenesis of BRCA1-associated tumors [15]. In ovarian cancer, immunohistochemical detectable overexpression of p53 is highly associated with presence of mutated, nonfunctional p53 [16]. However, Canevari et al. [7] suggest that p53 mutation is a late event in ovary carcinogenesis. In our study, the expression, mean percentage value of tumor cells presenting expression as well as intensity of immunostaining of p53 in BRCA1 mutation carriers were reduced in comparison to the control group. Lowest values have been noted in the study group patients with a history of breast cancer.

KAI1 is well known as a prostate cancer gene [9]. A report by Liu et al. [17] suggested that the downregulation of KAI1 expression may have a negative impact on survival in ovarian cancer. Also results by Houle et al. [9] suggest that the malignant progression of epithelial ovarian carcinomas is associated with downregulation and altered cellular localization of KAI1. Liu et al. [17] were unable to find any mutation of the KAI1 gene in primary or recurrent ovarian carcinomas except a missense polymorphism in codon 241. This finding confirms the observation that downregulation, rather than mutation, is a more common mechanism for the dysregulation of the KAI1 gene [17]. In human prostatic cancer, the expression of KAI1 was reported to be strongly correlated with that of p53, and the loss of both proteins was associated with poor survival [18]. This correlation was not found in ovarian carcinoma [8]. Houle et al. [9] observed a shift in protein localization of KAI1 from the membrane in grade 1 tumors to the cytoplasm in grade 3 tumors. They suggest that these changing patterns of expression from the membrane may be a mechanism by which tumor cells lose their adhesive properties during malignant transformation. In our study, expression of KAI1 in samples from BRCA1 mutation carriers seems to be downregulated again with lowest values in patients with a history of previous breast cancer. Also in our study, no correlation between KAI1 and p53 was found.

As the Nm23/NDP kinase-A gene was discovered in a murine melanoma metastasis model system, the correlation of its expression with tumor metastatic potential in actual human cancers was a subject of great interest [19]. However, breast carcinoma and melanoma are the two tumor types most studied in this context [19]; there are also reports in ovarian carcinoma [12, 20]. In study by Gao et al. [20], the expression of Nm23 RNA in human ovarian cancer cells was inversely related to metastatic behavior in the experimental animals and strongly suggested that the Nm23 expression correlates with reduced metastasis of ovarian carcinoma. Elevated Nm23 expression is also related to lower rates of lymph node metastasis and longer survival in endometrial cancer. Lower expression of Nm23 is an indicator of poor prognosis and may increase the risk of regional lymph node metastasis [21]. Adverse results reported by Srivatsa et al. [12] showed that expression of Nm23 is strongly upregulated in most epithelial ovarian cancer and suggest that Nm23 gene expression may have distinct if not opposite biologic functions in epithelial ovarian cancer and breast carcinoma.

## Conclusions

Observed lower expression of BRCA1, KAI1, NM23, and p53 may suggest the role of these proteins in carcinogenesis and poorer prognosis of hereditary ovarian cancer. However, no significant statistical differences between relatively small studied groups were found, the trends suggesting downregulation of these proteins in BRCA1 mutation carriers was reported. Lowest values were observed in patients with previous history of breast cancer of study group what might suggest that downregulation of studied proteins is combined with higher risk of carcinogenesis.
